# The "Alzheimer's disease signature": potential perspectives for novel biomarkers

**DOI:** 10.1186/1742-4933-8-7

**Published:** 2011-09-20

**Authors:** Sergio Davinelli, Mariano Intrieri, Claudio Russo, Alfonso Di Costanzo, Davide Zella, Paolo Bosco, Giovanni Scapagnini

**Affiliations:** 1 Department of Health Sciences, University of Molise, Campobasso, Italy; 2 Department of Biochemistry and Molecular Biology, Institute of Human Virology-School of Medicine, University of Maryland, Baltimore, MD, USA; 3 IRCCS Associazione Oasi Maria S.S., Institute for Research on Mental Retardation and Brain Aging, Troina, Enna, Italy

## Abstract

Alzheimer's disease is a progressive and neurodegenerative disorder which involves multiple molecular mechanisms. Intense research during the last years has accumulated a large body of data and the search for sensitive and specific biomarkers has undergone a rapid evolution. However, the diagnosis remains problematic and the current tests do not accurately detect the process leading to neurodegeneration. Biomarkers discovery and validation are considered the key aspects to support clinical diagnosis and provide discriminatory power between different stages of the disorder. A considerable challenge is to integrate different types of data from new potent approach to reach a common interpretation and replicate the findings across studies and populations. Furthermore, long-term clinical follow-up and combined analysis of several biomarkers are among the most promising perspectives to diagnose and manage the disease. The present review will focus on the recent published data providing an updated overview of the main achievements in the genetic and biochemical research of the Alzheimer's disease. We also discuss the latest and most significant results that will help to define a specific disease signature whose validity might be clinically relevant for future AD diagnosis.

## 1. Background

Alzheimer's disease (AD) is the most common form of dementia and age-dependent neurodegenerative disorder. It represents one of the major public health problems in our modern age and epidemiological investigations estimated that the amount of people with AD will rise to over 100 million by 2050 [1]. The main hallmarks of the disease are decreased number of neurons, formation of amyloid plaques and generation of neurofibrillary tangles which results in neuronal dysfunction. Although a definitive diagnosis of AD is not possible until autopsy, diagnostic tools to detect AD have improved considerably in recent years. Even though there was significant technological advance, improved antemortem AD diagnostic methods are still needed. To date, early diagnosis of AD is difficult, therefore an important challenge for the successful management of AD is the development of new tools to detect AD in its earliest stages which could predict the progression of the disease. In addition, it is necessary to translate neurobiological knowledge and biomarker research into clinical practice. In this perspective, there is a significant effort to discover novel candidate biomarkers that together with those well established will be able to improve the accuracy of diagnosis. Fortunately, there has been a significant progress toward the use of potent and modern methods which allow the concomitant measurement of several biomarkers but we are far to define and create a reliable diagnostic and prognostic profile.

According to the National Institute of Health, a biomarker is "a characteristic that is objectively measured and evaluated as an indicator of normal biologic processes, pathogenic processes, or pharmacologic responses to a therapeutic intervention" [2].

Nowadays there is still not an ideal biomarker able to improve differential diagnosis, track disease progression and measure treatment efficacy. This means that we have an urgent need to develop biomarkers that are sensitive and specific to AD pathology with positive and negative predictive value for the disorder [3]. In addition, it is crucial to understand the complex relationship between the different biomarkers. The main tests for biomarkers classes used in the diagnosis and prognosis of AD are positron emission tomography (PET) neuroimaging of β-amyloid (Aβ) protein deposition, magnetic resonance imaging (MRI) of volume hippocampus and other brain structures, quantification of abundance of proteins in cerebrospinal fluid (CSF) and blood (i.e., plasma and serum) especially with quantitative proteomics strategies, genotyping of genetic polymorphism and finally emerging approaches such as high throughput techniques, trascriptome analysis and next-generation DNA sequencing method.

Here, we will discuss the recent literature on the role of biological markers in AD, summarizing the status in this field and focusing on the most promising genetic and biochemical biomarkers.

## 2. Pathogenesis of Alzheimer's Disease and Amyloid Hypothesis

AD is the most frequent cause of dementia affecting more than 53 million people [4] worldwide and is often a multifactorial disease. The key clinical features of AD are progressive memory loss and cognitive decline. The disorder is histopathologically and morphologically characterized by deposition of extracellular plaques (Aβ42) and intracellular neurofibrillary tangles (tau) that are believed to play an active role in the neurodegenerative process of AD [5]. The amyloid cascade hypothesis articulated by J. Hardy is the central paradigm for the cause of AD and was prominent in the field of AD research [6]. The core of this hypothesis is an imbalance between the production and the clearance of Aβ in the brain [7]. The proteolytically derived product of amyloid precursor protein (APP) Aβ42 is the main constituent of the amyloid plaques. Aβ is generated by sequential actions of β-secretase and γ-secretase on APP through an amyloidogenic pathway and there are several truncated Aβ isoforms in the brain. The enzyme accountable for the β-secretase activity is β-site APP-cleaving enzyme 1 (BACE1) and an increased activity have been found in cases of prodromal AD [8,9]. Detailed description of the main causes of the AD have been extensively reviewed elsewhere [10] but it is essential to point out that the amyloid cascade hypothesis has less support today. Recently, many patients with severe AD showed no plaques at the post-mortem analysis and conversely the plaques may be found in the elderly without dementia [11]. It has now proposed that other forms of Aβ such as soluble Aβ oligomers cause AD [12] and interestingly it has been reported that the composition and levels of postmortem CSF biomarkers can differ from that of antemortem [13,14]. Another neuropathological hallmark of AD are inclusions of microtubule-associated protein tau that is the major constituent of neurofibrillary tangles. The abnormal hyperphosphorylation of tau causes dysfunctions in the axonal transport mainly due to the generation of insoluble aggregates during the formation of neurofibrillary tangles. Tau undergoes to post-traslational modifications [15] that may be involved in the pathology of the disease. In particular, it can be phosphorylated on multiple sites (to date 39 different sites verified) [16], which seems to be important in reducing the affinity of tau for microtubules [17]. As proposed by the amyloid hypothesis, changes in tau and resultant neurofibrillary tangles formation are triggered by toxic concentrations of Aβ. Despite the fact that the link between Aβ and tau is still unclear, several hypotheses have been proposed [18]. In order to develop new therapies and since it is possible that changes in phosphokinases could be involved in tau phosporylation, many protein kinase such as glycogen synthase kinase 3β (GSK3β), cyclin-dependent kinase 5 (CDK5), extracellular signal-related kinase 2 (ERK2), have been investigated as targets of treatments to reduce tau phosphorylation. Therefore, the cascade which leads to the formation of phosphorylated tau may contribute to synaptic and neuronal loss and preliminary work which aimed to reduce tau phosphorylation has shown that tau kinase inhibitors block neurofibrillary tangles formation in tau transgenic mouse but clinical testings are still under investigation [19].

AD has a widespread and complex molecular background, and different molecular changes occur in the disorder. Therefore, it has been suggested that there is no single molecular event which leads to AD but it is caused by different and multiple parallel mechanisms. Recently new findings supported the amyloid hypothesis [20], but also the tau pathology. In addition, important information and experimental evidence have emerged. One of these is the mitochondrial dysfunction with degeneration of mitochondria in neurons [21], but also phenomena such as inflammatory mechanisms, oxidative stress [22], vascular homeostasis, lipid metabolism [4] followed also by alterations in energy metabolism and antioxidant defense system.

## 3. Genetic of Alzheimer's disease

Genetically, AD is a complex and heterogeneous disorder which involves a combination of genes that need to be identified and validated. Additionally, the genetic risk of developing AD is around 70% [23] but familial AD accounts for less than 1% of the AD burden [7]. Generally, AD is divided into two forms: (1) early onset familial AD with Mendelian inheritance and (2) late onset AD named sporadic form, but it is necessary to emphasize that this historical dichotomy remains elusive for many aspects. However, among the genetic causes implicated in disease risk, three genes have been essential in our understanding of AD mechanism. Particularly, mutations in APP, presenilin-1 gene (PS1), and presenilin-2 gene (PS2) cause an autosomal dominant form of AD of young onset. These alterations are responsible for increase in Aβ generation or in the levels of Aβ42. Novel and fast high throughput approach such as DNA and RNA microarrays have led to the identification of multiple genes involved in different stages of AD [2], but it is important to avoid misidentification of false positive and to use these methods with great rigor. The main AD risk and familial genes are reported in table 1 and in addition to PS1 and PS2 that affect the accumulation of β-amyloid protein several other genes may have a role in the clearance or uptake of Aβ.

**Table 1:** Schematic Overview of Major Alzheimer's Disease Gene

Familial Genes	Locus	Functions
**APP**	21q21.3	APP gene encodes a membrane protein cleaved by secretase. Mutations in App locus causes autosomal dominant early onset AD and cerebroarterial amyloidosis.
		
**PS1**	14q24.2	PS1 is involved in APP processing and mutations can interfere the production of Aβ42 and to form plaques. Numerous alternatively spliced transcript variants encoding different isoforms have been identified for this gene.
		
**PS2**	1q42.13	Regulate APP processing as a part of the α-secretase complex. Familial mutations can change the production of Aβ42.
**Risk Genes**		
**APOE**	19q13.32	ApoE regulates the normal catabolism of triglyceride-rich lipoprotein constituents. APOE binds Aβ and it is involved in Aβ clearance. Subjects carrying the E4 allele have an increased amyloid burden.
		
**TAU**	17q21.31	The transcript undergoes complex alternative splicing and tau exists as six splice isoforms. The mutations can alter microtubule binding efficacy.
		
**DYRK1A**	21q22.13	DYRK1A is localized in the critical region of chromosome 21 and is involved in tau and APP phosphorylation. Firstly the activity is upregulated by Aβ and APP phosphorylation result in increased amyloidogenic processing with BACE interaction.
		
**GSK3β**	3q13.33	The overexpression of this gene may be relevant for AD. GSK-3 phosphorylates tau and presenilin-1, which are involved in the development of AD. The phosphorylation of tau leads to tangle formation and APP cleavage products can activate GSK3β resulting increased tau phosphorylation.
**New GWAS Genes**		
**CLU**	8p21.1	Clusterin is a chaperone molecule involved in clearence, aggregation and fibrillization of Aβ. It is associated with the progression of AD.
		
**PICALM**	11q14.2	Phosphatidylinositol binding clathrin assembly protein is associated with an increased risk of developing AD. PICALM plays a role in synaptic trasmission and may be involved in Aβ clearence. The protein is present in endosomes connected with AD.
		
**CR1**	1q32.2	This gene is a member of the receptors of complement activation (RCA) family, precisely the complement C3b protein, a key inflammatory protein activated in AD.
		
**BIN1**	2q14.3	This gene encodes several isoforms of a nucleocytoplasmic adaptor protein involved in endocytosis. BIN1 could have an effect on Aβ production and/or the clearance of Aβ.
		
**ABCA7**	19p13.3	This gene is a member of the superfamily of ATP-binding cassette (ABC) transporters and is highly expressed in brain, particularly in the microglia. ABCA7 inhibit β-amyloid secretion in cultured cells overexpressing APP.
		
**MS4A**	11q12.2	The genes in the MS4A cluster are locolized on chromosome 11 and encode proteins with at least 4 potential transmembrane domains but do not have specific function yet.
		
**CD2AP**	6p12.3	C2AP encodes a scaffolding molecule that regulates the actin cytoskeleton and is involved in the regulation of receptor-mediated endocytosis.
		
**EPHA1**	7q35	EPHA1 is a member of the ephrin receptor subfamily of the protein-tyrosine kinase family. It is implicated in synaptic development and plasticity but also axon guidance. Other functions have been proposed.
		
**CD33**	19q13.41	CD33 molecule belongs to the family of sialic acid-binding, immunoglobulinlike lectins. CD33 regulate the functions in the adaptive and innate immune systems both involved into the inflammatory reactions observed in the brains of AD patients.
		

Genes in genomic location are in according to Ensembl cytogenetic band

Recent studies indicate that the over-expression or some polymorphisms of phosphokinases, such as dual-specificity tyrosine phosphorylation-regulated kinase 1A (DYRK1A) contribute to an early onset of neurofibrillary degeneration, β-amyloidosis, neuronal loss [24] and this gene might be associated to the Aβ production and tau phosphorilation [25].

Apolipoprotein E (APOE) localized on chromosome 19 is the major genetic risk factor that account for the late onset AD; in particular the E4 variant (APOE4) of APOE gene is particularly important for sporadic AD but APOE4 is also associated with an earlier onset of the disease [26,27]. Three majors alleles (E2, E3, E4) are present on the chromosome but subjects carrying the E4 allele have increased amyloid deposition and recently it has been shown that the E4 allele affects memory and executive function [28]. APOE4 is the most reliable locus for AD but recent genome wide association studies (GWAS) have demonstrated other risk loci, in particular a study of 16 000 individuals showed association with loci of clusterin (CLU) and phosphatidylinositol-binding clathrin assembly protein (PICALM) genes [29]. Functionally, CLU is involved in clearance and aggregation of Aβ but also in Aβ fibrillization [30,31]. PICALM plays a role in clathrin-mediated endocytosis [32], synaptic transmission, and in the removal of apoptotic cells [33,34].

To date, several GWAS have been performed with several genes implicated in AD risk and progression rate and it is established by an emerging consensus that common genetic variation plays an important role in the etiology of AD. However, even though GWAS are critical in defining rare sequence variants that predispose to both early and late onset forms of AD, it is necessary to replicate genetic associations which would require case/control comparison of several hundreds of individuals.

The first considerable finding based on large scale sequencing technologies was GRB2-associated binding protein 2 (GAB2). GAB2 binds growth factor receptor-bound protein 2 (GRB2), which in turn can bind APP and both presenilins [35] but GAB2 is also associated with increased tau phosphorylation and its effect is most pronounced on carriers of the APOE4 allele [36]. In addition, new significant susceptibility loci are the complement receptor 1 gene (CR1) [37], a key inflammatory receptor protein activated in AD [38] and the bridging integrator 1 gene (BIN1) [39], which encodes an adaptor protein involved in receptor mediated endocytosis [40]. Interestingly, CLU, CR1 and PICALM have received much attention because supported by independent follow up studies [41].

The latest contribution to identify new susceptibility loci through GWAS was made by the Genetic and Environmental Risk in Alzheimer's Disease Consortium 1 (GERAD1) which showed ATP-binding cassette, sub-family A, member 7 (ABCA7) and membrane-spanning 4A (MS4A) gene cluster being two new AD associated loci. Further, the same study revealed genome-wide significance for CD2-associated protein (CD2AP), encoding ephrin receptor A1 (EPHA1) and Siglec-3 (CD33) that is a member of the sialic-acid-binding immunoglobulin-like lectins family and these three loci consisted in a follow-up indicative of association with AD [42].

Noticeably, other robust results come from the Alzheimer Disease Genetics Consortium (ADGC) that performed GWAS of late onset AD and accordingly we have now overall ten late onset AD susceptibility loci (APOE, CR1, CLU, PICALM, BIN1, EPHA1, MS4A, CD33, CD2AP and ABCA7) that enhance our understanding of the genetic architecture of AD [43]. Other genes that could be considered to have a potential relevance in the risk for AD are genetic variants involved in inflammation. Notably, interleukins (IL1A, IL1B, and IL6) are one of the strongest evidence of inflammatory agents that increase the risk of AD and significant polymorphisms are implicated in AD as demonstrated in several case/control studies [44]. It is widely accepted that genetic variation is important for the pathogenesis of AD and several researchers have tried to find out polymorphisms that may be related with it but convincing outcomes have not emerged yet. However, very recently Kennedy et al. reported that the brain-derived neurotrophic factor (BDNF) Val66Met (rs6265) polymorphism may have a genetic susceptibility mechanism for AD. These findings highlighted that the effect of BDNF Val66Met polymorphism confers risk of AD in an age-dependent manner [45]. In addition, genetic variants in the sortilin-related receptor (SORL1), involved in trafficking of APP, increases the risk of AD. Recent data of genetic associations suggest that changes in one of its homologs, the sortilin-related VPS10 domain containing receptor 1 (SORCS1) may affect the risk of AD [46]. It is also interesting to mention a new study that analyzed the region of APOE and of the adjacent gene translocase of outer mitochondrial membrane 40 (TOMM40) that are strongly associated with AD. Two poliymorphisms for APOE and TOMM40, respectively rs429358 and rs2075650, reached genome wide significance and showed association with Aβ and t-tau/Aβ ratios [47]. In order to investigate the genetic associations between the inflammatory mediator tumor necrosis factor-α (TNF-α) and AD, a detailed meta-analysis of polymorphisms in TNF-α was reviewed by Caruso et al. The study analyzed the association between 5 TNF-α polymorphisms (-850, -308, -863, -238, and -1031) and AD and suggested a specific genetic effect of the -850 single nucleotide polymorphism (SNP) on the AD risk [48].

Finally, it is important to provide further genetic biomarkers for the future but at the same time is vital to develop an accurate biomarker panel able to predict AD risk but also identify the pathways involved in the disorder and potential drug targets.

## 4. Biochemical markers for AD

The CSF is an optimal source of AD biomarkers because it is in intimate contact with the extracellular space of the brain and can reflect biochemical changes and metabolic processes that occur in the course of the disease. The diagnostic use of CSF for AD is difficult because collecting CSF requires an invasive treatment by lumbar puncture. Thus, other body fluids may be useful for AD diagnosis but also to monitor the progression of the disorder and to search for biomarkers. Metabolic products and proteins such as albumin, immunoglobulins, transferrin and α2-macroglobulin can be detect in plasma or serum but requires very sensitive methods because their total content especially in serum is very low.

### 4.1 Experimental approach to detect biochemical markers in AD

Here we will provide a brief overview on the main methods that are currently used to enhance our understanding of AD and develop a biomarkers panel that could support clinical diagnosis. All these methods have advantages and disadvantages but it is important to standardize the entire workflow including sample processing and instrument setup. There are several issues in the search of AD biomarker discovery but in order to develop novel diagnostic strategies to diagnose and monitor AD progression it is essential to complement 'omics' disciplines with other traditional methods. The major proteomics biomarker discovery methods are two-dimensional gel electrophoresis (2-DE) that provide high sensitivity and mass spectrometry (MS) based techniques.

For instance, the characterization of the human CSF proteome typically requires combined approach such as 2D-polyacrylamide gel electrophoresis (PAGE) with matrix-assisted laser desorption/ionization combined with time of flight MS (MALDI-TOF-MS) and liquid chromatography combined with electrospray ionization (LC-ESI-MS). Also the technology surrounding biomarker analysis in blood is developing rapidly and the increasing need of biomarkers in clinical trials has been faciliated by high throughput multiplex platform [49,50]. In addtion, there are several AD biomarker studies with surface enhanced laser desorption ionization (SELDI)-TOF-MS which provides an high throughput protein expression profile analysis [51]. Furthermore, most of proteomics methods for protein quantification are based on chemical labelling with stable isotope and different labelling strategies are available, such as isotope tagged relative and absolute quantitation (iTRAQ) [52], tandem mass tag (TMT) [53], isotope coded affinity tag (ICAT) [54] and isotope coded protein label (ICPL) [55]. In addition, antibody array provide an high throughput method to analize multiple biomarkers simultaneously. This assay, which was based on xMAP technology (Luminex, Austin, TX, USA) will replace enzyme linked immunosorbent assays (ELISA) in the measurement of the biomarkers.

Molecular imaging techniques improve diagnostic accuracy by reflecting brain function and single positron emission computed tomography (SPECT), PET, MRI provide relevant clinical results. PET using Pittsburg compound (PIB) allows the direct visualization of Aβ plaque burden in the brain when the patients are still alive. Structural MRI provide measures of brain atrophy [56] related to the degree of cognitive impairment [57]. Also the evolution of computational approaches could help the discovery of biomarkers that cause the disorder and the bioinformatics tools may reveal the high complexity of pathological mechanisms evidencing the protein networks that lead to AD.

### 4.2 CSF Biomarkers

The concentrations of several proteins in CSF reflects with good diagnostic accuracy the pathophysiological features of the disease. Particularly, there is an "AD signature" in the CSF [58] because the measurements of numerous studies have found that patients with AD have a marked increase in CSF levels of total tau (t-tau) and phosphorylated tau (p-tau) but characteristically show a reduction in Aβ42. Combining the various CSF markers may be useful to differentiate different forms of dementia [59] and to predict the conversion from mild cognitive impairment (MCI) to AD. In this perspective, Trojanowski et al. with targeted proteomic screen revealed novel CSF biomarkers that can improve the distinction between AD and non AD cases. Specifically, they identified some analytes including Aβ42, resistin and thrombospondin-1 that are associated with AD [60]. In 2010, the same author argued that Aβ42 is the most sensitive CSF biomarker for AD, with a sensitivity of 96.4% [61]. In addition, even though the combined analysis of Aβ42, t-tau and p-tau are approved in clinical diagnostic tools, some researchers have asserted that has less predictive value in preclinical testing [62].

Recent studies have been focused on the investigation of truncated Aβ isoforms and experimental data showed a novel pathway where short isoforms are generated by β-secretase and α-secretase and long isoforms are produced by γ-secretase [63]. Interestingly, the levels of Aβ (1-37, -38, -39, -40, -42) gave a 91% sensitivity and a 64% specificity in predicting the development of AD from MCI during a follow up study [64]. Other CSF biomarkers that may be valuable for AD are neuronal and synaptic proteins. Notably, visinin-like protein 1 (VLP-1), a calcium sensor protein expressed in high abundance in neurons, was found markedly increased in CSF of patients with AD [65]. Furthermore, an immunoassay for growth associated protein (GAP43), revealed increased levels of the protein in CSF from patients with AD [66]. AD specific changes in the CSF proteome have been found in many studies. For istance, variations that have been reported in AD include altered levels of α-1-antitrypsin, α-1b glycoprotein, APOA-I, APOE, retinol binding protein, vitamin D-binding protein, prostaglandin H2 D isomerase and transthyretin (TTR) [67-72]. Interestingly, the presence of APOE4 allele is associated with increased deposition of Aβ42 in the brain and decreased CSF level of Aβ42 [73].

Emerging evidence indicates that oxidative damage is involved in AD. For instance, lipid peroxidation affects the generation of F2-isoprostanes whose levels are often increased in the CSF of patients with AD [74]. Lipid peroxidation products have been found in brain, CSF and plasma from mammalian models with AD [75] and GWAS have identified new risk genes that are linked to lipid metabolism such as CLU also known as apolipoprotein J (ApoJ) [29].

Although there are several inflammatory biomarkers reported in CSF, none of them can be considered to have diagnostic or predictive value. However, some candidate CSF biomarkers of inflammation that showed increased levels in AD are TNF-α [76], monocyte chemotactic protein-1 [77], interferon γ-inducible protein 10, IL-8 [78], IL-6 [79], transforming growth factor-β (TGFβ) [80], vascular endothelial growth factor (VEGF) [79] and others but detecting changes of inflammatory molecules in CSF require larger scale of replication in cohorts of patients because of their low levels in the CSF.

Recently, using an unbiased proteomics approach (2D-DIGE LC-MS/MS), Fagan and co-workers identified four novel CSF biomarkers for AD (NrCAM, YKL-40, chromogranin A, carnosinase I). Notably, these markers can improve the diagnostic panel accuracy especially of Aβ42 and tau and may have the utility to better discriminate early symptomatic AD from cognitive normalcy but also other dementing conditions [81].

In a future perspective on the progress of CSF examination it might be useful to focus on post-translational modifications that have not been widely studied in CSF proteome studies. For example, phosphorylation is the most common tau post-translational modification described but other post-translational modifications have received much less attention and it is likely that additional modifications are required for the formation of tau aggregates. Various kinases and phosphatases regulate tau phosphorylation [82] suggesting their potential involvement in the development of new therapeutic drugs for AD.

Currently, in CSF several candidate biomarkers have been found but their applicability to improve the diagnosis of AD or to discover new drug targets have not established yet. However, numerous studies have been shown that the most promising biomarkers for AD in CSF include the combined analysis of Aβ42, t-tatu and p-tau that allows sensitive, reliable and specific diagnosis of AD identifying prodromal AD in cases of MCI.

### 4.3 Biomarkers in blood

In the last years many efforts were done to find disease specific and reliable blood biomarkers and different candidate such as α1-antitrypsin, complement factor H, α-2-macroglobulin, ApoJ and ApoA-1 have been proposed. However, the verification of changes in the levels of these molecules is difficult to verify in independent studies. In addition, it is significant to note that in the search for blood biomarkers correlated with AD the identification should be performed on the strength of accepted CSF markers, such as Aβ and tau related biomarkers. Furthermore, several documented evidence suggested that in the blood there is a protein signature related with AD but also in some way a transcript signature that might be relevant to predict and monitor the disease and increase reliability of the diagnosis. Although the study of the transcriptome as a potential source of biomarkers in blood could have many advantages the evidence of a transcript signature in blood are scant and its utility in AD diagnosis remains to be clarified. Table 2 lists the main biomarkers related to AD mentioned in this review.

**Table 2:** Main pathway and biomarkers AD related cited in this review

Pathway	Biomarker	Potential association with AD
**Signal transduction**	**GSK3β**	GSK3β integrates a variety of intracellular and extracellular pathways and appears to be increased in the AD brain. GSK3β is regulated by phosphorylation and is the major tau kinases.
		
	**CDK5**	Cdk5 plays a role in processes of neural development, synaptic signalling, learning and can influence tau phosphorylation indirectly via regulation of GSK3β.
		
	**ERK2**	The phosphorylation of tau by ERK2 induces tau to acquire biochemical properties of AD. ERK2 was detected in neurofibrillary tangles.
		
	**DYRK1A**	Dyrk1A is abnormally expressed in AD and recently it has been found to be associated with neurofibrillary tangles in sporadic AD.
		
	**PKC**	PKC has been implicated in memory mechanisms and is also involved in the processing of APP. The activators of PKC lead to increased processing of APP by the α-secretase pathway.
		
	**VLP-1**	Visinin-like protein 1 concentration is significantly altered in the CSF of AD patients and ia is associated with fibrillar tangles in AD brains.
		
**Oxidative stress**	**F2-isoprostanes**	Incresed levels of F2-isoprostanes are found in AD plasma and CSF.
		
**Inflammation**	**Interleukins**	Interleukins are consistently detected in the brains of AD and polymorphisms are implicated in AD. The activity in AD contributes to synaptic dysfunction and loss, and later, neuronal death.
		
	**TNF-α**	TNF-α has a central role in AD pathogenesis. The levels are increased in CSF and correlated with clinical deterioration.
		
	**C-reactive protein**	C-reactive protein has been found to be associated with AD in histopathological and longitudinal studies. It is associated with increased risk of AD.
		
	**α-1-antichymotrypsin**	α-1-antichymotrypsin participates in the inflammatory cascade of AD and enhances the formation of amyloid-fibrils.
		
	**α2-macroglobulin**	α2-macroglobulin has an important role in AD etiopathology. The main ability is to mediate the clearance and degradation of Aβ.
		
	**Homocysteine**	Hyperhomocysteinaemia is a risk factor for AD and mental decline.
		
	**ICAM-1**	ICAM-1 is expressed on cerebrovascular endothelium and neuritic plaques in brain of AD patients and seems to be implicated in the process of neuro-degeneration.
		
	**VCAM-1**	Abnormal levels of VCAM-1 levels have been found in individuals with AD as well as other cell adhesion molecules.
		
**Lipid metabolism**	**Total cholesterol**	High concentration of serum cholesterol is associated with increased risk of incident AD.
		
	**APOE**	APOE E2, E3, and E4 alleles alter the likelihood of developing AD and cerebral amyloid angiopathy.

In 2007, with a combined multivariate analysis of 18 plasma signaling and inflammatory proteins (e.g. IL-1α, IL-3, TNF-α) Ray and colleagues identified a profile that was indicative of AD and predicted AD in patients with MCI [50]. Plasma Aβ was examined in different studies but provided opposing data because Aβ binds several plasma proteins resulting in epitope masking and other analytical interferences [83]. The role of inflammation with microglia activation is believed to play a role in AD pathogenesis but the presence of inflammatory markers in serum or plasma is unclear. Inflammatory molecules, such as IL-1β, TNF-α, IL-6, C-reactive protein, a1-antichymotrypsin showed contrasting results [84]. Seshadri and colleagues in the Framingham study observed that high levels of peripheral blood mononuclear cell (PBMC) of the inflammatory cytokines such as IL-1 or TNF-α are associated with an increased risk of developing AD [85].

Noteworthy, Teunissen et al. evaluated 29 serum biomarkers for inflammation, cholesterol and homocysteine metabolism, and brain specific proteins. This panel including IL-6 receptor, cysteine and cholesterol demonstrated to be a suitable combination to discriminate AD from controls [86]. Several evidence have documented that cholesterol metabolism plays a role in AD [87]. Total serum cholesterol may be a marker of AD because high concentration of serum cholesterol is associated with increased risk of incident AD [88]. In the brain monocytes migrate through the blood-brain barrier (BBB) interacting with specific cell adhesion molecules (CAM). Several CAMs on monocytes are activated during the inflammatory and neurodegenerative response. Therefore, these could be useful as biomarkers in AD. Humpel and co-workers reported that monocytic ICAM-3 and P-selectin are significantly reduced in AD patients [89].

A growing body of evidence demonstrates that plasma concentration of vascular cell adhesion molecule-1 (VCAM-1) and intercellular adhesion molecule-1 (ICAM-1) are increased in AD. In addition, endothelial vasodilatory such as endothelin (ET-1), adrenomedullin (ADM), and atrial natriuretic peptide (ANP), as well as sphingolipids are altered in mild AD and MCI suggesting sensitivity of these biomarkers for early detection and diagnosis [90].

Another potential biomarker important for AD diagnosis because involved in tau phosphorylation, is the protein kinase C (PKC). The PKC function is involved in memory processes in animal models [91] and appears altered in red blood cells and lymphocytes of AD patients [92]. By inhibiting GSK3β, PKC reduces tau phosphorylation and neurofibrillary tangles formation [93] representing a potential target for the development of disease modifying drugs.

In summary, according to the diagnostic and prognostic point of view, the most promising markers in AD diagnosis to be associated with Aβ and tau include α1-antitrypsin, α-2-macroglobulin, apo lipoproteins and TTR.

Conclusively, it seems clear that the combinations of different biomarkers can improve the diagnosis of AD but the markers in the CSF and blood are not enough and other molecules are abnormally processed in AD.

Overall, by analyzing 13 different brain regions of AD affected patients Korolainen et al. demonstrated changes in 93 proteins in association with the disease [94]. These proteins showed quantitative differences and/or post-translational modifications in cognitive impairment in early and late AD. According to the gene ontology (GO) classification these proteins are involved in oxidation-reduction (12), glycolysis (8), transport (8), metabolic processes (7), protein folding (6), the response to unfolded proteins (5) and cell proliferation (5). 56 of them are cytoplasmic, 28 mitochondrial, 20 nuclear and 16 cytosolic proteins. Finally, three of them are synaptic proteins (synaptosomal-associated protein-25 (SNAP-25), synaptotagmin and syntaxin-binding protein) which present altered expression or modification.

## 5. Concluding remarks

We have highlighted different biomarkers that may be important for the detection and differential diagnosis of AD. It is clear that many questions remain to be answered especially because we know only few evidence about the relationships between these biomarkers and the development of the disease. Although the combined analysis of Aβ, t-tau and p-tau can be used to diagnose AD, it is important to characterize the core of biomarkers involved in the prodromal phase of AD but also in the presymptomatic stage of the disease. For this purpose only the combination of several biomarker derived from CSF and other body fluids will be efficacious to define a specific signature of AD. The measurement of AD biomarkers in the CSF or by structural and functional imaging and genetics methods improves diagnostic accuracy but also the prospect for blood based biomarkers is attractive. Taken together these tools have revealed the effective importance of molecular mechanism that contribute to pathological changes and neurodegeneration. For instance, these processes are oxidative stress, overproduction of reactive oxygen species, changes in ubiquitination, reorganization in the cytoskeletal proteins, production of misfolded protein and many others. Furthermore, it is important to implement common protocol and to standardize the ultrasensitive analytical methods for experimental design and generation data.

## Competing interests

The authors declare that they have no competing interests.

## Authors' contributions

All the Authors drafted the manuscript and approved the final manuscript.
